# Application of Operational Research Techniques in Operating Room Scheduling Problems: Literature Overview

**DOI:** 10.1155/2018/5341394

**Published:** 2018-06-13

**Authors:** Şeyda Gür, Tamer Eren

**Affiliations:** Department of Industrial Engineering, Faculty of Engineering, Kırıkkale University, 71450 Kırıkkale, Turkey

## Abstract

Increased healthcare costs are pushing hospitals to reduce costs and increase the quality of care. Operating rooms are the most important source of income and expense for hospitals. Therefore, the hospital management focuses on the effectiveness of schedules and plans. This study includes analyses of recent research on operating room scheduling and planning. Most studies in the literature, from 2000 to the present day, were evaluated according to patient characteristics, performance measures, solution techniques used in the research, the uncertainty of the problem, applicability of the research, and the planning strategy to be dealt within the solution. One hundred seventy studies were examined in detail, after scanning the Emerald, Science Direct, JSTOR, Springer, Taylor and Francis, and Google Scholar databases. To facilitate the identification of these studies, they are grouped according to the different criteria of concern and then, a detailed overview is presented.

## 1. Introduction

Hospitals, whose production output is service, have begun to take strategic steps for the services they provide due to increased health requirements and the competitive environment. Therefore, hospital management needs to reduce costs and improve financial assets. Operating rooms earn two-thirds of hospital incomes and also constitute for about 40% of hospital expenses [1]. From this point of view, operating rooms account for the largest share in terms of both income and expenditure. For this reason, the increase in the productivity of operating rooms has an important influence on the financial and ultimate ethical performance of hospitals. As a result, operating rooms constitute the most interesting and attractive areas in hospitals [2]. With these performance improvements, service quality and patient satisfaction are increasing in direct proportion.

The operating room scheduling problem is treated as a special branch in optimization problems. For the past four decades, researchers have been cautiously focused on planning and scheduling studies to achieve goals such as performance and productivity in the operating room. In the literature, researchers have developed a wide range of approaches to the solution process by identifying the problem. Solutions have been offered to these problems by considering different performance criteria.

This study aims at analyzing in detail the studies in the literature related to the operating room scheduling problem in hospitals. It also shows the criteria that are based on healthcare plans. In addition, this work provides up-to-date and general information about the planning and scheduling of health service systems. It explores how services systems have taken steps against the increasingly high costs of medical technology and how they use their resources efficiently. We present work that explicitly includes this information and contributions made to this area. Moreover, with the contributions obtained from the research carried out, this area is reflected such that it can be easily understood by readers who want to do further research. In addition, bringing together thoroughly the literature examined in detail provides a better definition of the studied subject. When the literature review was conducted, operating room scheduling and planning keywords were searched for in the Emerald, Science Direct, JSTOR, Springer, Taylor and Francis, and Google Scholar databases. The results of the examination allowed 170 studies to be compiled.

When the literature is examined, it is seen first that there are limited number of literature review studies related to operating room scheduling and planning. Cayirli et al. [3] reviewed the literature on the problem of scheduling outpatient treatment in hospitals and examined 70 studies. They aimed at presenting the modeling approaches in detail. They narrowed the broad scope of health services according to search criteria and purposes. Cardoen et al. [4] presented a detailed analysis of 115 studies by reviewing the literature on operating room scheduling. They categorized their work according to these features, drawing attention to certain features encountered during the scheduling phase. Thus, they prepared a study that makes it easy to access more sophisticated and frequently searched for search criteria. Guerriero and Guido [5] analyzed 130 research works related to the application of operational research in surgical planning and scheduling studies and examined the results of the problem types encountered in the solution approaches. Detailed information is given related to the steps taken in the management of the operating room and management in hospitals more widely, and relevant optimization studies on this topic are evaluated.

Unlike other studies in the literature, all of the studies reviewed in this study date from the year 2000 and later. This is because of the increase in the annual budget that operating rooms were consuming at the end of the 1990s, which has become the focus of both hospital administrators and researchers. Besides financial assets, various performance measures and approaches have been developed by considering the problem dimension that they deal with from different angles. When all of the research done is taken into consideration, it appears that the vast majority was carried out after 2000. Moreover, since the development of technology has also caused changes in the working structures of organizations, current studies are focusing on more complex problems. For these reasons, in this study, we limited the research dimension to both analyzing the contributions of the new approaches developed and increasing accessibility. However, the studies that have been investigated have been examined according to different perspectives and are presented to the reader. Considering that hospitals are one of the key areas of operation research, we focus this research on the scheduling and planning of operating rooms. Information on the efficient and effective use of operating rooms has been given by conveying the strategic situations considered in planning and scheduling studies. The different perspectives discussed in the study provide the reader with immediate access to the information they seek. This study, which facilitates direct access to information and includes up-to-date research, is significant in different ways, examining operating rooms from both managerial and procedural perspectives.

The review structure of this study includes subject headings according to the criteria specified. This study, structured according to more specific and descriptive characteristics, is divided into 7 sections.  Section 2, on patient characteristics, includes examining the literature according to the patient's elective (inpatient or outpatient) or nonelective (urgency) status.  Section 3 examines performance criteria, waiting times, postponed operations, utilization of the operating room, financial assets, and preferences. In Section 4, the research methodology is based on the analytical method employed and the evaluation techniques applied in the solution process. In Section 5, the state of uncertainty is examined according to the stochastic or deterministic states of the studies examined. In Section 6, the applicability of the research, the data used in the studies, and the applications are examined. In Section 7, the planned strategic steps to be taken in the operating room are reported. Each section that is identified for analysis includes the detailed structure of the works and the list of works done, as well as briefly mentioning the related terminology.

## 2. Patient Features

Existing studies on operating room scheduling and planning in the literature are divided into two major groups, as elective and nonelective patients. The elective patient group is able to preplan and does not involve any ambiguity and variability. The nonelective patient group is also known as emergency patients. This is a group of patients who need urgent intervention because they face life-threatening risks. This phrase is used to show the urgency and priority of the clinical interventions. Due to the uncertainty of this group's structure, they do not form part of the planning of surgeons beforehand, but instead arise unexpectedly. The first priority is given to this emergency group of patients, and then, the other patient groups are included in the planning process [3]. The nonelective patient group constitutes a large part of the surgical demand and takes priority. Scheduling and planning for this type of patient group in hospitals is considered a difficult task. Accepting such operations in hospitals requires them to consider both reserving existing capacity and taking into account uncertainty at the same time. The other group of patients relates to previously planned operations [6]. In the literature, the elective patient group has a greater share of scheduling and planning than the nonelective patient group. In the vast majority of studies, researchers distinguish between the two groups of patients in which their work is located, although they do not fully describe the elective patient group. Even though in most of the studies on scheduling and planning of the operating room, the financial assets of the hospital are reduced, and revenues are increased, Nouaouri et al. [7] did research on how hospitals should use their existing resources in areas where unusual conditions such as disasters or catastrophic damage could occur. In such cases, victims need to be referred to hospitals in nearby regions for urgent treatment. In the face of such urgency, hospitals have developed a reactive approach that focuses on maximizing human survival by ignoring financial assets. They recommend reorganizing the operation plan if necessary. The problem of operating room scheduling involves many uncertainties due to its structure. Because of this, most studies make certain assumptions in the solution process, without considering these uncertainties. When these uncertainties arise, some researchers favor rescheduling. van Essen et al. [8] considered the uncertainties in surgical times and also plans that are interrupted due to the arrival of emergency operations. They developed a decision support system for this problem and determined the best-corrected plan for the operating room. Looking at the results, they observed that fewer operations were canceled with this decision support system.

If the nonelective patient group is considered, the hospital should respond to this emergency as soon as possible. Erdem et al. [9] presented an approach with a mixed integer linear programming method for rescheduling elective patients in the event of emergency operations. As a distinguishing feature from similar studies, the cost of rejecting urgent health conditions, which has a critical impact on the hospital in an emergency, is included in the model structure. They gained a broad perspective through the use of a genetic algorithm which allows the model to provide the most appropriate solutions under difficult scenarios. Thus, they achieved a superior solution quality for problem sets containing high patient loads.

There is a significant impact on the hospital's policy-setting capacity from the need to allow emergency surgical situations while planning and scheduling elective patients. Marques et al. [10] pointed out two conflicting goals when scheduling an elective patient group. They used a metaheuristic approach with integer linear programming with the aim of reducing waiting lists by rationalizing resources. Khanna et al. [11] noted the difficulties experienced in the surgical scheduling of the elective patient group. They developed a predictive-based methodology for planning processes in order to gain a general viewpoint. They created a template that represents the utilization of the operating room by conducting a retrospective analysis of estimated workload information and waiting lists. ShahabiKargar et al. [12] used regression analysis to estimate the duration of operation procedures for elective patient groups. Putting the focus on the utilization of the operating room offered an algorithm for making more accurate predictions for the manager. Jung et al. [13] proposed an integrated approach to help to make a balanced plan with the need to react to needs arising during operating room planning. This approach, which consists of a three-step process, allows rescheduling for emergency patients after elective patients have been allocated to the operating room and resources. In their work, Neyshabouri and Berg [14] developed a formulation that considers the intensive care unit (ICU), which is one of the other departments related to the operating room. They also combined a simulation model and a formulation to understand the level of risk associated with the proposed surgical plans. They relieved the obstacles that could be experienced in the operating room with a robust two-step optimization method to avoid the uncertainties of the duration of surgery.  Table 1 presents the studies according to patient features.

From Table 1, it is seen that researchers focus more on the elective group of patients. The nonelective patient group is overlooked more because of the difficulty of transferring it to the models created. When this situation is examined, it is stated by researchers that it is difficult to plan the operating room as the degree of uncertainty in the problem increases. However, it is easier to associate the elective patient group with the expected financial assets in the scheduling process. The studies in Table 1 divide the patient group into two. Unlike these studies, the study by Zonderland et al. [111] focuses on the semiurgent group of patients. Semiurgent patient groups, besides the other emergency groups, are defined as patient groups whose symptoms include such cases as spinal fractures with or without minimal neurological symptoms. This patient group was considered with the Markov decision chain. Many other authors, on the contrary, in their work, see as a source of motivation the degree of uncertainty resulting from the nonelective patient group and indicate that they privatize their work. At the same time, a significant number of studies do not specify the patient group during the scheduling and planning processes. From a general point of view, the lack of clear definition of operating room scheduling and planning problems in terms of patient features suggests that many studies are inadequate.

In the literature examined for the two groups of patients, the elective patient group is frequently preferred by researchers for convenience in the solution process. In these studies, surgeons identify the operations and they will perform at the beginning of the week and plan the timing for these selected patient groups. Often, in these studies, they aim at balancing the utilization of the operating room and reducing waiting times for patients on the waiting list. There are many assumptions for planning in this patient group. The uncertainty of patients' arrival times is ignored by most studies, such as 1, 9, 10, 15–20, and 70–72. In addition, no studies that planned simultaneously for these two groups of patients were found [4]. Future studies can take these situations into account by developing new algorithms to address this deficiency in the literature. Because of the priorities with which emergency cases are regarded, they must be operated on the day of admission. When these cases arrive at the hospital, an operation in the elective patient group is canceled when there is no appropriate operating room. After these cancellations, surgeons are then working overtime. In further studies by researchers, with new models or algorithms, they may consider extra costs due to overtime and cancellations, overtime capacity constraints, and the inclusion of both elective and nonelective patient groups without cancellations. Planning can be done to reduce assumptions along with various uncertainties such as the time of arrival of the patients, the duration of the surgical procedure, and considering all the organizational and technical constraints. When evaluating both elective and nonelective patient groups, the waiting time of patients, as well as the effect on the workload of the staff and hospital, should be considered.

## 3. Performance Criteria

Various performance measures are used in evaluating operating room planning and scheduling problems in the literature. While these performance measures customize the structure of the problem, they also limit the size. As the number of evaluated criteria increases, the problem structure becomes more difficult and complicated. Individual performance measures have been distinguished, including waiting time, utilization, patient postponement, cost, and so on. The studies examined in Table 2 are classified according to these performance criteria.

Table 2 contains several studies that include other performance measures. Looking at these studies from a broad perspective, it is actually seen that researchers have taken different approaches for planning of hospital organizations. In the studies reviewed, researchers often considered the balanced utilization of operating rooms and the reduction of costs. The complexity and interactions of all these factors are a source of problems for hospital managers, who are looking for efficient and effective utilization of operating rooms and want to keep the patient/staff satisfaction level high. Within this context, they are searching for the most appropriate operating room scheduling and planning. Researchers should increase the criterion level they consider for future studies. Although not particularly emphasized, there should be a focus on the balanced operation of the other parts of the operating room that are integrated. The compatibility between actual situations and schedules that are made without considering these units can be examined. In addition, patient postponement or rejection, which is among the performance criteria, can result in serious damage to the hospital both materially and reputationally. However, this measure has not been adopted very much in the literature. Researchers should analyze the relationship between these criteria for future studies. Since it is very difficult to evaluate all these criteria at the same time, they should make a plan that takes these criteria into consideration, as the outcome of the relationship is most likely to contribute to the hospital. Then, as a result of the planning they have done, they should evaluate the performance of the given data, whether this is actual data or specific probability distributions. The degree of satisfaction of patients on long waiting lists in hospitals directly affects the motivation of the healthcare institutions in terms of both material and morale [16]. The group on the waiting list is divided by researchers into two groups, namely, surgeons and patients. The importance of the satisfaction of the surgeons is emphasized as much as the degree of satisfaction of the patients. During the planning of the operating room, they offer a combination that allows surgeons to reduce their waiting times. They touch on the relationship between the duration of operations and the waiting times of the surgeons. The accuracy of the time estimates of these operations describes the quality of operating room scheduling.

Utilization, which is shown as another performance criterion, has been set as the objective by many studies in the literature. In addition, researchers handled the utilization criterion separately in terms of operating room sections. A large majority focus is in particular on the utilization rate of operating rooms. Because of the large financial asset represented by operating room utilization rates, even small changes in the schedules have effects on various overheads such as overtime pay at the hospital. Many studies in the literature have developed different approaches to the effectiveness of the utilization of operating rooms and have noted the impact of both overuse and underuse. From this point of view, they emphasize that the efficiency of operating room use should be kept at the maximum level in the balance of these two cases. They propose a hierarchical approach as an alternative to the difficulty of computation [17], which relates to the utilization of operating rooms because of the distribution of operations balanced between surgeon groups.

One important factor that can make hospital organizations more effective is the increase in costs in the health services. The benefit of utilizing the most efficient operating room capacity cannot be ignored. Planning processes involving basic objectives such as the effectiveness of resources in hospital organizations are dimensioned as strategic, operational, and tactical. van Oostrum et al. [22] developed a model that meets the requirements for utilization of the operating room by addressing planning at the tactical level with the solution approach they offer. Augusto et al. [65] focused their work on the daily planning of operating rooms, where various constraints were reflected in the model they set up. They helped management by improving the utilization of operating rooms. Tan et al. [60] reached goals in their solution approach to reduce variability in bed occupancy rates, as well as in the operating room effectiveness, which varies with over- and underuse.

Another criterion that is as important as the utilization of the operating room is the utilization of intensive care units. The intensive care unit capacity, measured by the number of beds on these units, is a source of concern for hospitals. Kim et al. [75] focused on improving the performance of intensive care units effectively and efficiently to make a positive contribution to healthcare in their model.

This criterion, which is included in the performance criteria as patients postponed, indicates the quality of the service given to the patient. Addis et al. [67] guaranteed the quality of service provided at a certain level of performance. They developed a punishment function to prevent deferrals and delays due to postponements. Likewise, they presented an optimization approach with a block scheduling strategy [33], which provides a penalty function to avoid as many patient postponements as possible. In order to assess the models in terms of solution quality, the number of patients operated on, the waiting times, and the delays experienced were examined.

One of the most common goals in operating room scheduling problems is the expected performance in terms of financial assets. In general, an operating room planning and scheduling problem is indirectly affected by the cost criterion, even when other goals are considered. That is why, in fact, this criterion is among the cornerstones of healthcare units. Meskens et al. [152] developed a model that considers constraints on material resources encountered in real life. With an efficient algorithm, they created a schedule that allows for the efficient utilization of the operating room and the surgeon. Baesler et al. [143] focused on a scheduling approach aimed at maximizing hospital revenues by addressing the plans in the organizational structure at strategic and tactical levels.

Another performance criterion is the preference criterion which is adopted as an aim by researchers in the process of scheduling and planning the operating room. Van Huele et al. [158] created a formulation that relieves both surgical and nonsurgical constraints. In planning that considers the surgeon's preferences, the effects of these preferences are examined. Xiang et al. [145] considered surgeons' experiences of scheduling problems in their work. They developed a balanced planning and scheduling approach based on the inclusion of certain surgeons in some operation groups. They analyzed the effectiveness of their algorithm with this preference option for the surgeons.

## 4. Techniques Used in Solution Processes

Operating room planning and scheduling processes affect the entire hospital organization. These processes are increasingly complicated by the inclusion of areas such as the intensive care unit (ICU), or the PACU, which are other parts of the operating room. But it is considered beneficial from a strategic point of view to improve the overall process. Given the work involved in these facilities, the researchers' results highlight the extent to which the performance quality increases. At the same time, when the uncertainties of these facilities are not overlooked, it seems that the long-term effect for the hospital is in the positive direction.  Table 3 contains the structure of studies in the literature in terms of solution techniques. While most studies address the operating room on its own, other studies are available that incorporate simultaneous solution approaches integrated with these facilities. In addition to these, recently reported operating room scheduling studies are also related to health services, although they are seen to be integrated with different fields.

Looking at Table 3, it is observed that improvements have been made in the planning and scheduling processes through the integrated studies carried out in recent periods, although a great majority of studies have shifted to practice where the operating room is considered alone. The analysis of the consequences of these integrations is also an important gateway to the work to be undertaken in the coming years. In the real world, the operating room is integrated with rest of the hospital such as the PACU and ICU. This makes the planning process very difficult. Often there are disruptions from planning that is not done correctly or that does not balance these integrated parts correctly. These disruptions have negative consequences, such as postponement or rejection of patients, an increase in surgeons' waiting time, or prolonged preparation and cleaning time. This gives the hospital both extra costs and patient/staff dissatisfaction. Researchers can conduct studies that analyze the impact of these negative outcomes on the patient, as opposed to planning work in general. The overall performance of the operating room can be evaluated. Later, as a result of this study, the most important factor affecting hospitals in the negative direction can be investigated. If this factor is most relevant to a certain department, plans can be made to improve that department. Focusing on the details of the studies, it is seen that some of the criteria such as utilization of the operating room, reduction of patient waiting lists, cost, and similar criteria are taken together. Problems that are integrated with different units focus more on specific functions.

The main objective was to improve the utilization rates of the units that they integrate within these special functions. The studies under the other heading in Table 3 are mostly studies where nurse units are considered together. The special situations of the nurse units and their relations with the operating room are reflected. Martinelly et al. [34] developed a model proposal that shows the relationship between the operating room and nurse management, and the number of operating rooms, number of nurses, and overtime concepts. When the main purpose of the study is examined, an integration that represents two different management areas is seen with a flexible model understanding. They established an operating room scheduling model that both plans for nurses and at the same time considers resource constraints. The model incorporates nurse restrictions that make the process even more difficult for already complex operating room processes. However, the results show that there is no relation between the number of operating rooms and the number of nurses. It is stated that there is an inverse relationship between the number of nurses and the amount of overtime work. This, in fact, means that the validity of the nurses in the integration of both management areas is small. In the operating room scheduling and planning literature, there are methodologies that use a specific analysis and solution technique.  Table 4 lists these methodologies and what they focus on.

**Figure 1:**
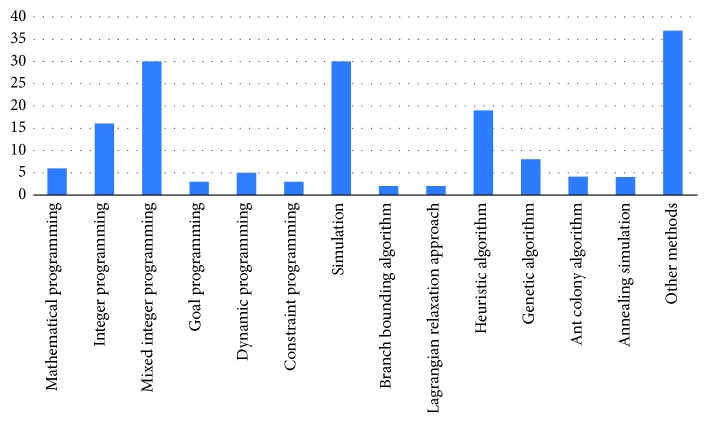
Solution techniques.

Table 4 presents a perspective on the analysis of problems. It seems that there are different suggestions that can help reduce the difficulty of calculation when there are slight changes in the structure of the problems. In the use of solution methodologies where performance measures are effective, many researchers are discussing how to approach uncertainty as the amount of uncertainty increases and the resulting effectiveness of the established model structure. From the work done, it is seen that researchers go to the solution process by using the advantages of each method. In fact, these solution methods require various assumptions to be made in the problem. Every algorithm or model that has been developed gives very effective results day-by-day in the process of operating room scheduling and planning. However effective they are, these results are not enough and must be continuously improved, and the solution area expanded. Researchers can leverage the power of constraint programming to create mathematical or logical representations of existing constraints in the problem. With constraint programming, many solution areas can be found in the definition cluster and the most suitable one can be selected within the solution area. This allows the evaluation of different values in the solution process. Moreover, in order to obtain satisfactory results in a short time, heuristic methods can be used, and a solution approach can be developed for queuing models to nonelective patient groups. Beliën et al. [24] optimized the bed occupancy rates with the optimization system they have set up, allowing the same specialist surgeons to concentrate on the same operating room. It is seen that the results of the calculations produced successful plans based on these two objectives.  Figure 1 expresses the location of the solution techniques in the literature visually.

From Figure 1, it can be seen that more simulation and mathematical models are used in the solution process of the problem studied. There is also diversity under the headings mentioned as other methods in the solution process. Researchers have brought different perspectives to the solutions of problems through different techniques. It is difficult to produce alternative solutions to the challenge of the operating room scheduling problem and to obtain high quality results from these solutions. They have helped to improve the functioning of the operating room [87], which successfully reflects the expression power of the solution approach they have proposed to solve these difficulties. They are focused on the daily planning of the operating room with the constraint programming method. In addition, human and material constraints that reflect the surgeon's preferences are included in the model. When the results are examined, it appears that the solution method they use is an ideal tool for competing goals. Meskens [152] compared their constraint programming method with a mixed integer programming model, another optimization tool, to examine their effectiveness in real-life problems. They considered various constraints in their work and presented the advantages and disadvantages of both models.

When we look at the work done recently, we prefer integrated methods rather than using a single solution technique, due to the different external factors that make the problem structure more difficult. Researchers have aimed at increasing the quality of the solution by using integrated methods. Furthermore, the analysis of the scenarios with test data is performed with the presented simulation approaches. If it is emphasized that these analyses support the implementation results, this can be interpreted as indicating that the approaches are successful.

## 5. Uncertainty Status

One of the biggest problems encountered in the planning and scheduling of operating rooms is that there is too much ambiguity due to the structure of these problems. Many researchers have considered various assumptions for the production of correct programs and for the development of contributions to hospital organizations. When the literature is examined, it focuses on the uncertainties in patient arrivals and operation times. When we look at the literature on stochastic studies, unpredictable arrivals, especially of nonelective patient groups, have various effects. These sudden occurrences in planning have a negative effect for both surgeons and patients. At the same time, uncertainties in the duration of surgical operations are critical for operating room planning. Operations that exceed the predicted duration affect not only the start time of the next operation in the program but also all the day's other operations. These late start-ups affect the shift times in the planning, right up to the last working hour of the day, and result in staff overtime costs.  Table 5 lists the stochastic and deterministic approaches.

When detailed analyses of the studies are carried out, it can be seen in detail in Table 5 that the uncertainties in times and arrival times affect waiting lists and the utilization of resources. In addition, the uncertainties in the margin of the contribution to the hospital structure, which will keep the expected high financial cost, also indicate that operating rooms affect the utilization capacity. However, attention should also be paid to the difficulties that may arise from failure of the hospital's medical equipment. In the literature, future studies should give more importance to this source of uncertainty, so that improvements in the quality of the solution can be realized by researchers, because this is an important problem that needs attention when it affects the starting times of operations. By increasing the number of studies to reduce the negative effects of such uncertainties in the coming years, researchers will be able to make significant contributions to both real-life practices and the literature. Due to the difficulties in the solution process of these uncertainties in terms of their structure, it seems that stochastic studies are not very useful. Also, in the literature, the capacity requirements in emergency situations, the arrival times of these emergencies, and the duration of operations are usually neglected. These neglected situations should be addressed through stochastic studies by researchers.

## 6. Applicability of the Study

When the literature is examined, a comprehensive test is applied to analyze the performance of the developed models. For these experimental tests that demonstrate to what extent goals can be reached, a significant amount of data entry is required. Looking at Table 6, in the studies listed, it is seen that performance analysis of most of the studies is done using theoretical data. The data used in these studies are divided into two groups; the actual data/set of theoretical data is obtained either at random or with a certain probability distribution. However, even if data sets from real-life problems are used in studies, most of the developed approaches are not reflected in the real application. In this case, researchers should concentrate on the reasons for conflicting ideas between the application phase and the model they are developing.

Table 6 gives an analysis of studies of the use of real solution data and the different solution techniques in Table 4. The results obtained from the experimental tests on the developed models show that the operating rooms need to be more balanced according to the current utilization conditions and help to create proposals for flexible use at less cost. In hospitals, which are regarded as service units, the planning that is prepared for the operating room may include several possible mishaps. Therefore, it is seen as beneficial by researchers to perform short-term real applications of the studies. However, the point of view of hospital administrators, in hospital organizations that already have a difficult and complicated structure, is that the sudden application of these studies may complicate problem. Banditori et al. [30] focused on reducing the number of patients on the waiting list by considering a plan for each day of the month. At the same time, the aim was to avoid cost increases caused by this waiting and the negative effects that might be experienced on satisfaction levels. Accordingly, a set of solutions was produced. As with every study, these studies have limits. The authors who conducted experimental simulation tests using the real-life data mention the difficulties that models can experience in the planning process due to the 1-month planning horizon.

Researchers should use more of the experimental sets obtained from real data to assess the performance of the planning and schedules. Planning and schedules need to be applied in real life to allow the healthiest performance measurement. Hospital administrators who allow this can increase hospital efficiency with the performance values obtained as a result of these plans. In addition, researchers can comment on which points in the schedules they test with actual data need to be developed or which points they should concentrate on. With the actual data used, they can show the robustness of the model they have built and the extent to which it can be put into real practice. Since the studies prepared under various assumptions neglect many sources, it is difficult for managers to perceive the positive potential of real applications. On the contrary, if it is judged to be very difficult or even impossible to take all the assumptions into consideration, hospital organizations need to take strategic steps to support such work, because in reality no hospital can make all these assumptions.

## 7. Planning Strategies

**Figure 2:**
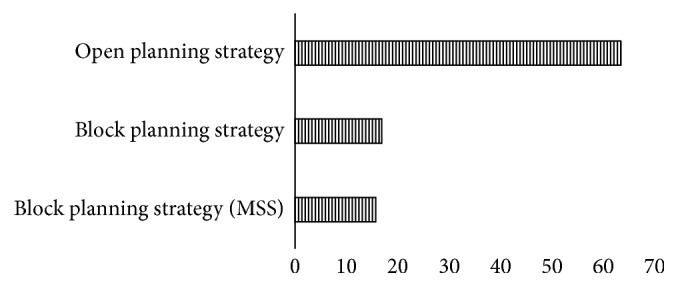
Planning strategies.

In hospitals that provide healthcare, managers want to maximize the yield from the utilization of the operating rooms through a variety of strategic steps. This has led to different strategic plans. Hospital administrators have planned strategic steps in operating rooms, including open planning strategy, block planning strategy, and modified block planning strategy. The studies in Table 7 are listed according to these strategic steps. In Figure 2, the distributions of the studies are shown visually.

When we look at Table 7 and Figure 2 together, it is seen that the open planning strategy is most common. The block planning strategy is divided into two parts: block planning with Master Surgical Scheduling (MSS) and block planning strategy only. It appears that this distinction has emerged from the different situations in which researchers handle their work from a managerial point of view. When the sections under the block planning strategy are examined together, it is seen in many studies that researchers think that it is time and space that should be reserved for surgical specialties. Researchers commonly choose one of two parts reserved for planning strategies in the scheduling process. However, unlike most studies, Liu et al. [118] addressed the open planning and block planning strategy together. They also developed a metaheuristic algorithm to solve this problem. The open planning strategy allows surgeons to be assigned to appropriate operating rooms with appropriate time. When an empty schedule is considered, it is assumed that patients will be received on a first-come-first-serve basis, taking into consideration their arrival times. For schedules created in the block planning strategy, the same day of the week, the same time zone, and the same operating room are stored in the service of a particular surgeon or specialist. With this strategy, it is necessary to adjust the appropriate operating room at the hours when the operating rooms are open.

The modified block planning strategy is a process of reconfiguring operations that are not in the previously constructed blocks for unused time. This is described as flexible planning because it requires the reorganization of the initial construction schedules. In future studies, using block planning strategy, the preferences of surgeons can be given more importance and the efficiency of these plans can be increased. Also, with block scheduling, certain times within the surgeons' working hours can be left empty. Thus, it is both separate from the surgeons' rest time and makes it easier to allocate a suitable operating room in the event of an emergency. This can reduce the delays that can be experienced during the preparation and cleaning periods between operations as well as the patient waiting time that is caused by these conditions.

When these studies are examined, it is seen that they allow better use of the surgeon's time, and at the same time, prevent delays that may occur due to extra preparation time for operations requiring different surgical expertise in the operating room. This has made block planning strategies the focus of researchers. The block planning strategy, which is associated with the main surgical scheduling problem, defined as the allocation of operational resources to surgical groups, is being considered in many studies. In the literature examined, there are 16 main surgical scheduling studies [20, 22, 24, 25, 27, 30, 31, 40, 49, 53, 56, 60, 61, 88, 150, 151]. Mannino et al. [27] considered the problem of estimating demand levels in creating the main surgical schedules and then aimed at stabilizing patient tail lengths and reducing the maximum overtime. In their work, they introduced new approaches to help users with strategic planning. Addis et al. [33] aimed at reducing waiting times for patients by assuming a block planning strategy. In the study, particular attention was paid to ensure that the operating room capacity is balanced in terms of surgical expertise. In this article, which is also integrated with the master surgical schedules, the waiting patient set is allocated to the operating rooms.

While studies of open planning strategies were popular during the 1960s, today different strategies for increasing productivity continue to be developed. But nowadays, too, there are many studies that use open planning strategy in order to avoid the difficulty and complexity of calculation. In [26], no specific time is reserved for any particular surgeon with the open planning strategy. As is the case in most studies, the main point of this study was to increase the efficiency of the utilization of the operating room.

## 8. Conclusion

This study examined 170 planning and scheduling studies related to operating rooms scanned in the databases of the Emerald, Science Direct, JSTOR, Springer, Taylor and Francis, and Google Scholar. The contributions of these studies to the literature and the reader were assessed and the points they emphasized were identified. While evaluating the goals that the studies want to accomplish, the technical structure was examined. For the analysis of the studies, a systematic structure was established in this study so as to make it easy to focus on what readers want specifically to investigate. In addition, by comparing the studies, it can be easily seen which point of study the work has taken forward. Clear lists were created with the tables presented to improve the accessibility of the findings.

It is seen that optimization methods are generally preferred in the studies about the planning and scheduling of the operating room, with the aim of providing the best result within the solution process. At the same time, efforts are also made to avoid complicating the model due to the various constraints encountered in real life, and the solution area is created within these frameworks to improve the process. Researchers have emphasized the need to balance the resources available and improve the effectiveness of staff in order to optimize the utilization of operating rooms, which hospital administrators see as the most critical part. Optimal utilization of operating rooms is possible when assessed with different performance measures. Even though indirectly, it is difficult to reflect these interrelated criteria together, so the solutions are proposed under many assumptions.

Another problem that complicates the solution process of the problems is that there is too much uncertainty. In the studies, patients were separated into two groups, ignoring the uncertainty of their arrival times. In studies dealing with a nonelective group of patients, it was noticed that other operations were postponed or canceled when an unplanned case occurred in the created schedules. Delayed operations cause both surgeons and other staff to work overtime and reduce the level of satisfaction by increasing patient complaints. It was seen that the efficient utilization of operating rooms was obstructed when workers on overtime exhibited stressful behavior in the working environment. This situation is negatively reflected as an extra cost to hospital managers. In the type of problem that researchers are dealing with, it is necessary to pay attention to such situations. As a primary goal, efforts to improve the financial asset represented by the operating room should be increased. The effect of such factors on this goal should be observed, and the contributions made in this direction must be reported in the literature. Stochastic studies that take into account sources of uncertainty should be increased and concentrated on stochastic efficiency durations.

Throughout the review, important points about scheduling and planning of operating rooms are emphasized. Emphasis has been placed on the points of interest in the studies, and points distinguishing these studies from each other are listed in tables. Relevant terminologies are given in the subject headings so that the reader is first informed about this. This type of problem, which attracts interest in optimization problems, has recently become the focus of researchers, and the various approaches developed are presented in this study. It is noticed that every work done is a guide to other works and constitutes different study approaches for future researchers. It is thought that it is necessary to encourage these schedules and plans, which are mostly done manually, to be more systematic with these developed approaches. Since these studies are not yet fully implemented in hospital organizations, their actual effects on the operating rooms and personnel are not known, even if actual performance analyses are being performed. With this literature review, these points have been considered and the review is focused only on the scheduling of the operating rooms. The factors that are considered in the operating room scheduling studies and which distinguish these studies from each other constitute the boundaries of this review.

The content of this study takes into account the limitations and factors that affect only operating room schedules, and it is believed that this contributes by helping readers to access the information directly. When we look at the studies from a wide perspective, it is seen that the solution process becomes more difficult as the features added to the problem dimension increase. Researchers studying this issue have preferred to limit the problem dimension. It is considered that researchers need to analyze the deviations in these goals while realizing the goals they set. When flexibility is allowed in the structure of the model, it is necessary to emphasize how the result changes. Considering the lack of attention to these aspects in the literature, it is predicted that new approaches for future studies can be developed. It is thought that it is possible to measure the performance of these allowed deficiencies by using various techniques.

## Figures and Tables

**Table 1:** Patient features.

Elective patient group	[1, 6, 8–100]

Nonelective patient group	[6, 7, 9, 13, 21, 35, 48, 52, 74, 90, 97, 99, 101–110]

**Table 2:** Performance criteria.

Waiting time	Patient	[6, 16, 17, 19, 20, 25, 30–33, 37, 46, 54, 57, 67, 74, 77, 78, 82, 83, 90, 91, 93, 97, 98, 100–102, 104, 112–119]
	Surgeon	[77, 114, 120, 121]

Utilization	Operating room	[1, 8, 11, 15, 17, 20, 22, 25, 26, 28, 32, 36, 37, 38, 42, 46, 48, 54, 60, 62, 65, 66, 68, 70, 73, 76, 78, 82, 84–87, 89, 92, 95, 98–100, 102, 103, 105, 106, 109, 110, 112, 114, 117, 118, 120, 122–135, 136–142]
	ICU (intensive care unit)	[14, 22, 24, 38, 41, 42, 48, 51, 60, 61, 65, 69, 70, 75, 78, 80, 82, 85, 110, 122, 124, 143, 144]

Overtime	Operating room	[6, 15, 20, 21, 26, 27, 29, 34, 43, 46, 53, 60, 62, 69, 73, 76, 84, 95, 96, 101, 104, 117, 120, 124, 125, 145, 146, 137]
	ICU	[6]
	PACU (postanesthesia care unit)	[117]

Completion time		[21, 65, 66, 86, 91, 143, 147, 148]

Patient postponement/rejection		[33, 67, 90, 94–96, 111, 118, 119, 146]

Financial asset		[1, 21, 23, 25, 35, 36, 40, 44, 45, 52, 61–63, 69, 79, 80, 81, 87, 92, 93, 98, 111, 112, 114, 117, 121, 126, 139, 149–156]

Preferences		[15, 39, 72, 78, 87, 144, 145, 157]

Humanitarian goals		[7, 15, 43–45, 55, 83, 84, 104, 106, 108, 152–154, 158]

Others		[7, 9, 11–13, 18, 22, 23, 27, 29, 31, 34, 35, 42, 49, 50, 51, 55, 56, 58, 59, 63, 64, 67, 71, 72, 74, 88, 97, 99, 103, 105, 107, 113, 116, 120, 129, 130–132, 136, 144, 148, 150, 151, 158, 159, 160–170]

**Table 3:** Status of the operating room.

Only the operating room		[1, 6, 7, 10, 11–19, 21, 22, 24, 26–29, 31–33, 35–37, 39, 42–47, 50, 52–57, 59, 60, 62–64, 66–68, 70–74, 76–79, 82, 83, 87, 89, 90, 92–94, 99, 101–103, 105–107, 109, 111–116, 120–123, 125–130, 132–135, 137, 138, 142, 143, 145–147, 149–155, 159–163, 165, 167, 168]

Integrated operating room	PACU	[9, 49, 84, 85, 117, 131]
	ICU	[25, 30, 38, 41, 48, 49, 51, 61, 65, 69, 75, 80, 81, 124, 144]
	Others	[20, 23, 34, 40, 58, 86, 88, 104, 108, 110, 136, 148, 157, 158, 164, 166, 169]

**Table 4:** Solution techniques.

Mathematical Programming	[17, 33, 34, 47, 60, 90, 92, 125, 136, 142, 164]

Integer programming	[8, 10, 23, 25, 28, 32, 41, 49, 52–54, 95, 110, 113, 114, 122, 140, 149, 156, 159]

Mixed integer programming	[1, 9, 13, 21, 24, 27, 30, 36, 40, 44, 56, 58, 62, 66, 68, 70, 71, 78, 84, 85, 86, 93, 100, 102, 104, 126, 128, 134, 138, 144, 152, 157, 162]

Goal programming	[15, 16, 42]

Dynamic programming	[23, 149, 160]

Constraint programming	[87, 137, 152, 166]

Simulation	[6, 14, 20, 21, 30, 31, 35, 38, 49, 51, 55, 58, 61, 73, 76, 77, 83, 91, 95, 96, 97, 102, 105, 106, 116, 118, 119, 131, 133, 143, 145, 153, 158, 161, 163, 167]

Branch bounding algorithm	[61, 121]

Lagrangian relaxation approach	[45, 65, 148]

Heuristic algorithm	[7, 13, 18, 26, 37, 54, 57, 59, 73, 85, 103, 109, 112, 113, 115, 123, 128, 147, 162, 118, 94, 156, 140, 98, 141]

Genetic algorithm	[9, 19, 62, 82, 89, 124, 131, 135, 154]

Ant colony algorithm	[29, 46, 145, 164]

Annealing simulation	[24, 51, 77, 143]

Other methods	[6, 11, 12, 22, 38, 39, 43, 46, 48, 50, 63, 64, 67, 69, 72, 74, 79, 80, 81, 88, 92, 99, 101, 107, 108, 111, 117, 120, 127, 129, 130, 132, 139, 146, 150, 151, 155, 161, 165, 168, 169, 170]

**Table 5:** Uncertainty status.

Deterministic	[1, 9, 10, 13, 15, 16, 17, 18, 19, 20, 23, 25, 26, 28, 29, 31, 32, 34, 36, 37, 41, 42, 44, 47, 49, 50, 53–60, 62, 64–72, 78, 79, 82, 83, 85–89, 100, 102, 112, 115, 124, 125, 127, 129, 137, 144, 145, 147, 150, 152, 157, 159, 170]

Stochastic	[6, 13, 14, 20, 21, 22, 24, 27, 33, 35, 38, 40, 48, 51, 52, 61, 63, 73, 76, 77, 80, 84, 90, 92, 95, 104–110, 114, 117, 126, 130, 131, 138, 143, 155, 160, 162]

**Table 6:** Application of studies.

Not tested		[35, 50, 57, 70, 75, 87, 144, 152]

Test data	Theoretical data	[1, 6, 7, 9, 14, 19, 21, 23, 25, 26, 28, 29, 32, 34, 36, 39, 41, 44–49, 51, 52, 58, 61, 63, 65, 67, 71, 72, 74, 79, 80, 83, 85, 86, 91, 94, 103, 105, 107, 109, 112, 114, 116, 117, 120, 127, 129, 131, 133, 135, 136, 139, 145, 147, 153, 157, 158, 160, 161, 166]
	Real data	[10, 11–13, 15–18, 20, 22, 24, 27, 29–31, 33, 37, 38, 40, 42, 43, 53–56, 59, 60, 62, 64, 66, 68, 69, 73, 76–78, 82, 84, 88, 93, 95, 97, 100, 102, 104, 106, 110, 113, 115, 121–126, 128, 130, 132, 134, 138, 141–143, 146, 149, 150, 151, 154, 156, 159, 162–165, 168, 170]

**Table 7:** Planning strategies.

Open planning strategy	[1, 6, 9, 10, 12, 13, 16–18, 21, 23, 26, 28, 29, 32, 34, 35, 37, 38, 43, 44, 46, 48, 52, 54, 55, 58, 59, 62, 63, 65, 66, 68, 69, 73–77, 80, 81, 83–87, 101, 104, 106–110, 112, 118, 124–127, 130, 139, 147, 149, 152, 157, 161]

Block planning strategy	[15, 19, 20, 22, 24, 25, 27, 30, 31, 33, 36, 40, 41, 49–51, 53, 56, 60, 61, 67, 78, 88, 89, 95, 102, 103, 105, 114, 116, 118, 129, 145, 150, 151, 159, 170]
